# Effects of treatment with montelukast alone, budesonide/formoterol alone and a combination of both in cough variant asthma

**DOI:** 10.1186/s12931-022-02114-6

**Published:** 2022-10-10

**Authors:** Fang Yi, Chen Zhan, Baojuan Liu, Hu Li, Jianmeng Zhou, Jiaman Tang, Wen Peng, Wei Luo, Qiaoli Chen, Kefang Lai

**Affiliations:** Guangzhou Institute of Respiratory Health, State Key Laboratory of Respiratory Disease, National Clinical Research Center for Respiratory Disease, National Center for Respiratory Medicine, The First Affiliated Hospital of Guangzhou Medical University, 151 Yanjiang Rd., Guangzhou, 510120 Guangdong China

**Keywords:** Cough variant asthma, Montelukast, Budesonide/formoterol, Cough, Eosinophilic airway inflammation

## Abstract

**Background:**

Whether cysteinyl-leukotriene receptor antagonists (LTRAs) have a similar antitussive effect to inhaled corticosteroids and long-acting β2-agonist (ICS/LABA), and that LTRA plus ICS/LABA is superior to LTRAs alone or ICS/LABA alone in treating cough variant asthma (CVA) remain unclear. This study aimed to investigate and compare the efficacy of montelukast alone, budesonide/formoterol alone and the combination of both in the treatment of CVA.

**Methods:**

Ninety-nine CVA patients were assigned randomly in a 1:1:1 ratio to receive montelukast (M group: 10 mg, once daily), budesonide/formoterol (BF group: 160/4.5 μg, one puff, twice daily), or montelukast plus budesonide/formoterol (MBF group) for 8 weeks. The primary outcomes were changes in the cough visual analogue scale (VAS) score, daytime cough symptom score (CSS) and night-time CSS, and the secondary outcomes comprised changes in cough reflex sensitivity (CRS), the percentage of sputum eosinophils (sputum Eos%) and fractional exhaled nitric oxide (FeNO). CRS was presented with the lowest concentration of capsaicin that induced at least 5 coughs (C5). The repeated measure was used in data analysis.

**Results:**

The median cough VAS score (median from 6.0 to 2.0 in the M group, 5.0 to 1.0 in the BF group and 6.0 to 1.0 in the MBF group, all *p* < 0.001), daytime CSS (all *p* < 0.01) and night-time CSS (all *p* < 0.001) decreased significantly in all three groups after treatment for 8 weeks. Meanwhile, the LogC5 and sputum Eos% improved significantly in all three groups after 8 weeks treatment (all *p* < 0.05). No significant differences were found in the changes of the VAS score, daytime and night-time CSSs, LogC5 and sputum Eos% among the three groups from baseline to week 8 (all *p* > 0.05). The BF and MBF groups also showed significant decreases in FeNO after 8 weeks treatment (*p* = 0.001 and *p* = 0.008, respectively), while no significant change was found in the M group (*p* = 0.457). Treatment with MBF for 8 weeks significantly improved the FEV_1_/FVC as well as the MMEF% pred and decreased the blood Eos% (all p < 0.05).

**Conclusions:**

Montelukast alone, budesonide/formoterol alone and a combination of both were effective in improving cough symptom, decreasing cough reflex sensitivity and alleviating eosinophilic airway inflammation in patients with CVA, and the antitussive effect and anti-eosinophilic airway inflammation were similar.

*Trial registration* ClinicalTrials.gov, number NCT01404013.

**Supplementary Information:**

The online version contains supplementary material available at 10.1186/s12931-022-02114-6.

## Background

Cough variant asthma (CVA) is a phenotype of asthma that presents solely or predominantly with cough and airway hyperresponsiveness but not with wheezing or dyspnea [1]. Our multicentre survey showed that CVA was the most common cause of chronic cough, accounting for more than one-third of cases [2]. CVA shares a number of pathophysiological features with bronchial asthma, such as airway hyperresponsiveness, eosinophilic airway inflammation and airway remodelling [1, 3–5]. Moreover, cysteinyl leukotrienes (CysLTs) have been reported to play a major role in the pathogenesis of asthma, including chronic inflammation of the airway, bronchoconstriction and airway remodelling [6, 7]. The sputum level of CysLTs has been shown to be increased in CVA as well as in classic asthma patients [8, 9].

Similar to classic asthma, the cough guideline recommends inhaled corticosteroids (ICS) as the initial treatment for patients with CVA and that β2-agonists could be combined with ICS [10, 11]. As a cysteinyl-leukotriene receptor antagonist (LTRA), montelukast was recommended as an alternative or add-on treatment for asthma due to its efficacy in reducing airway inflammation and controlling asthma. Meanwhile, the ERS and ACCP cough guidelines suggested considering a therapeutic trial of a leukotriene inhibitor after an incomplete or a failure response to ICS treatment in patients with CVA [10, 11]. Whether there is a difference between LTRAs and ICS/LABA in treating CVA remains unclear. In addition, although the efficacy of LTRAs in the treatment of CVA has been reported in a few studies, the sample sizes were small. Dicpinigaitis et al. found that zafirlukast significantly improved the cough score and cough reflex sensitivity (CRS) in 8 CVA patients; they concluded that zafirlukast can relieve the cough of CVA by decreasing CRS [12]. Another placebo-controlled study found that montelukast significantly decreased the cough frequency and total daily cough symptom score of 8 patients with CVA [13]. Nevertheless, a change in airway inflammation was not observed in these studies. Recently, a prospective study conducted by Takemura et al. showed that montelukast significantly decreased the cough VAS score, cough sensitivity and sputum eosinophil count, whereas lung function remained unchanged, indicating that the antitussive effect of montelukast on CVA might be attributed to its anti-inflammatory effects [14]. The mechanisms of LTRAs in the treatment of CVA need further study. Furthermore, limited studies have investigated the prognosis, e.g., cough relapse and wheezing development, of patients with CVA after LTRAs treatment.

Thus, in this study, we aimed to investigate and compare the efficacy of montelukast alone, budesonide/formoterol alone and the combination of both in the treatment of CVA in terms of cough symptom, cough reflex sensitivity, airway inflammation and lung function.

## Methods

### Study design and subjects

**Fig. 1:**
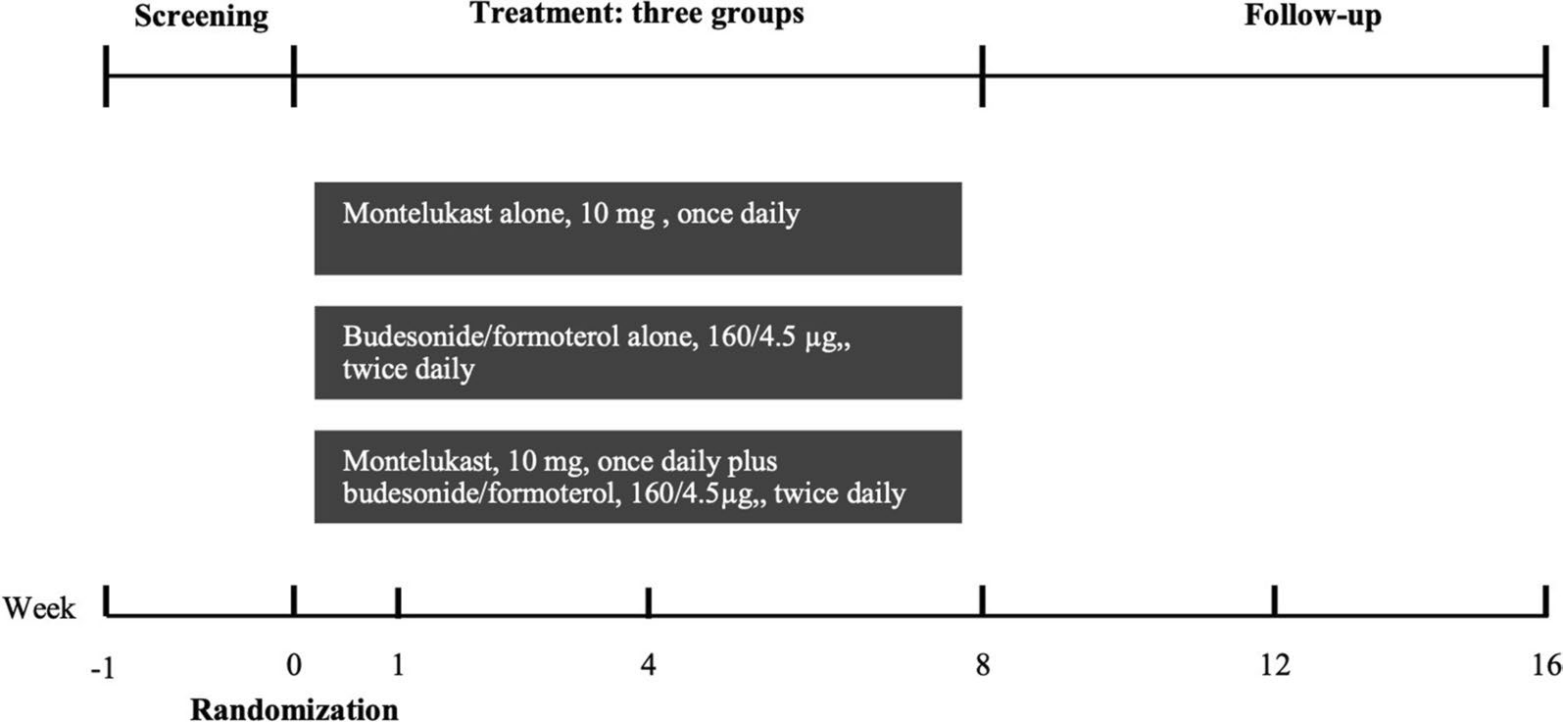
Summary of study design and visits

This study was an 8-week, randomized, open-label, parallel-group study to compare the effects of montelukast alone, budesonide/formoterol alone or a combination of both in CVA patients (Fig. 1). Patients with chronic cough who attended the specialist cough clinic in the First Affiliated Hospital of Guangzhou Medical University were screened. The causes of chronic cough were diagnosed via a validated management algorithm according to the Chinese national guidelines on the diagnosis and management of cough [15]. We included patients with CVA with a prolonged cough lasting more than 8 weeks, with no substantial abnormalities on chest radiograph and a positive result of greater than 12% FEV_1_ reversibility after short-acting bronchodilator use or a positive methacholine provocative test. Patients were excluded if they were current smokers, had quit smoking less than 1 year before enrolment in the study, had a history of upper or lower respiratory tract infection within 4 weeks, had complications of other serious respiratory diseases, had received anti-asthmatic treatment including corticosteroids (e.g., oral corticosteroids, ICS and ICS/LABA), LTRAs et al. in the previous 4 weeks, or were pregnant or lactating women.

The study was approved by the Ethics Committee of the First Affiliated Hospital of Guangzhou Medical University. All patients provided written informed consent. The trial was registered with ClinicalTrials.gov, with number NCT01404013. The study was supported by Merck & Co., INc. The funder did not play a role in study design, data analysis, data interpretation, or writing of the report.

### Procedures

**Fig. 2:**
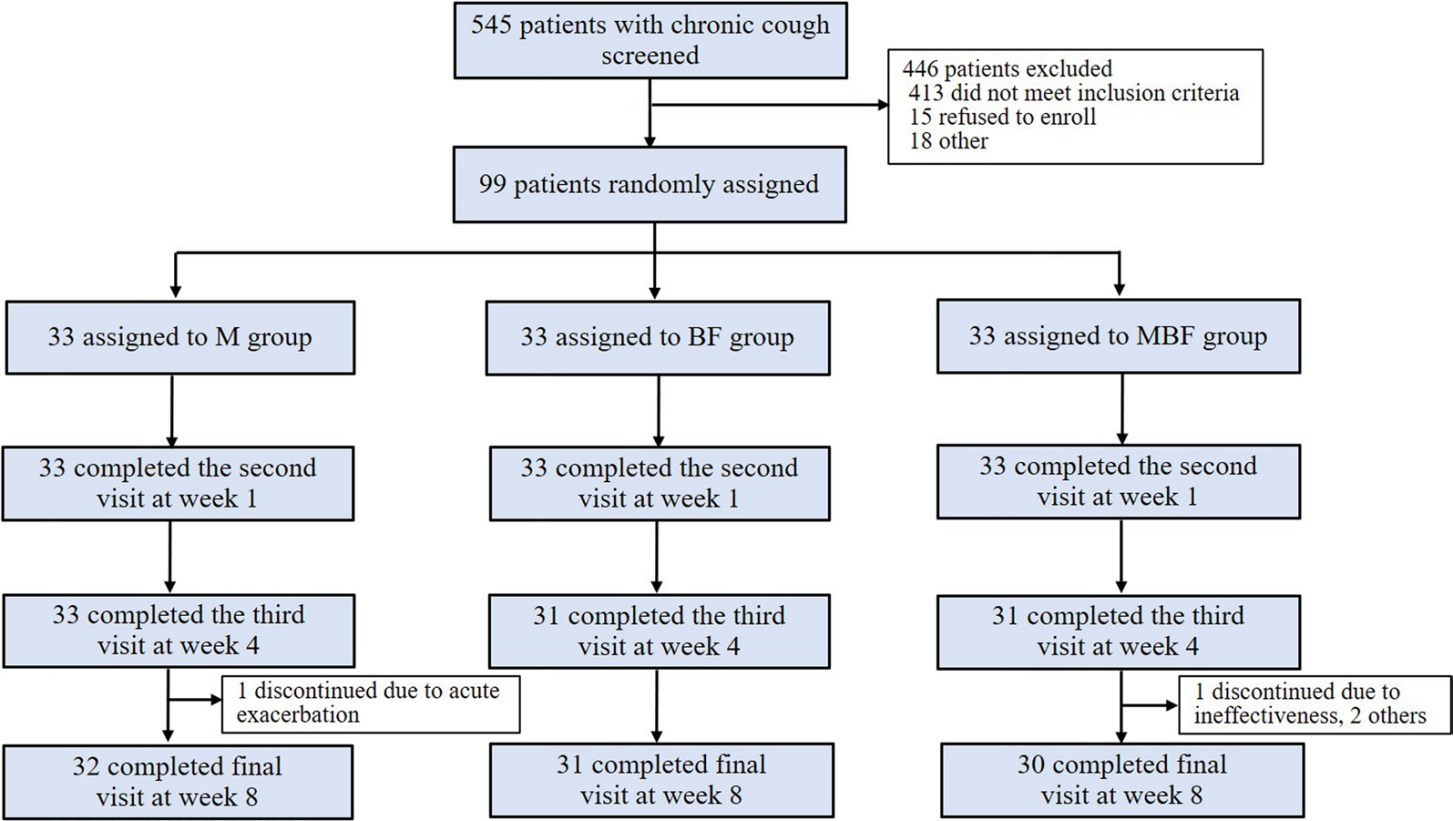
Trial profile. Full analysis set included all randomly assigned patients who had taken at least one dose of study medication and provided at least one baseline and one post-baseline primary endpoint observation during the treatment period. M: montelukast alone; BF: budesonide/formoterol alone; MBF: montelukast plus budesonide/formoterol

The patient screening assessment included medical history, a physical examination, vital signs, chest X-ray, routine blood test, spirometry and bronchial challenge test, sputum induction, and clinical questionnaire. After the screening assessment, eligible patients were randomly assigned (1:1:1) to a sequence of montelukast alone (M group: 10 mg, once daily), budesonide/formoterol alone (BF group: 160/4.5 μg, one puff, twice daily) or montelukast plus budesonide/formoterol for 8 weeks, followed by a further 8-week follow-up. The randomization schedule was computer generated, using a permuted block algorithm, and randomization numbers were allocated to patients. Both the investigators and patients were aware of the treatment allocation. The trial profile is shown in Fig. 2. The cough Visual Analogue Scale (VAS), cough Symptom Score (CSS), fractional exhaled nitric oxide (FeNO), sputum induction for differential cells, spirometry and bronchial challenge test, capsaicin cough challenge, and routine blood test were performed at the follow-up visit. After 8 weeks of treatment, the patients were followed up for another 8 weeks by telephone. If patients experienced a cough relapse or progressed to wheezing during the follow-up period, they received a consultation in the clinic and data were recorded specifically regarding of the development of their symptoms. Patients with exacerbations were managed according to the cough guidelines [15, 16].

Cough severity was assessed with the VAS and CSS. The cough VAS is a 10 cm scale for patients to describe the severity of cough, with a higher score indicating a more severe cough. The CSS is a two-part questionnaire scoring including daytime cough and night-time cough, respectively, in which each part score ranges from 0 to 5; 0 indicates no cough and 5 indicates distressing coughs for most of the day or preventing any sleep during the night.

Cough reflex sensitivity was tested in accordance with our previous report [17]. The lowest concentration of capsaicin that caused 5 or more coughs was recorded as the cough threshold (C5). Eventually, the cough reflex sensitivity was presented as the logarithm of C5 (LogC5). Sputum was induced and processed as described in our previous study [18]. The FeNO measurement was performed in accordance with the standard procedure described in the ATS and ERS recommendations [19]. Briefly, the subjects were instructed to inhale deeply via a mouthpiece and then exhale with a constant flow (0.05 L/s) for 10 s. Spirometry and bronchial provocation tests were performed by Jaeger Master Screen (Germany) according to the ATS recommendations [20]. The provocative dose of methacholine causing a 20% fall in FEV_1_ (PD_20_) was adopted as the marker for airway hyperresponsiveness. Airway hyperresponsiveness was defined as a PD_20_ < 12.8 µmol. Atopy was defined as the presence of a positive skin reaction to any allergen or an increased level of serum IgE ≥ 0.35 KU/L.

### Outcomes

The primary outcomes were the cough improvement as reflected by changes from baseline in the 10 cm cough severity VAS score, daytime CSS, and night-time CSS after 8 weeks of treatment. The secondary endpoints included the changes in the LogC5, the percentages of sputum eosinophils (Eos%) and FeNO, which were evaluated at week 4 and 8. In addition, lung function and percentages of blood eosinophils were measured at baseline and week 8. Additionally, the onset time of cough improvement reported by the patients themselves regarding of the first time from taking the medication to the time that the cough started to show improvement was recorded.

### Statistical analysis

The primary analysis used data from the intention-to-treat population, which included all randomized patients who took at least one dose of the study drug. We did not impute missing data. Statistical analysis was conducted via STATA 15.0 and SPSS (version 18.0). Age, cough VAS scores, daytime and night-time CSS, LogC5, sputum Eos% and blood Eos% were expressed as the median (interquartile range, IQR); FeNO was expressed as the geometric mean ± SD; the FVC% pred, FEV_1_% pred, and FEV_1_/FVC ratio were expressed as the mean ± SD; and change in values of the VAS, CSS, LogC5, sputum Eos% and FeNO from baseline to week 8 were expressed as the mean (95% CI). The primary and secondary outcomes were analyzed using a mixed model repeated measure. One-way ANOVA or the Kruskal–Wallis rank test were used to compare the change of each index. A *p* value < 0.05 was considered statistically significant.

The study was a noninferiority trial on the decrease in cough severity at 8 weeks as assessed by the difference in the score measured at week 8 week and baseline among the groups. A difference of 1.5 in the VAS score (0–10) can be considered clinically significant [21, 22]. Hence, we decided to set the zone of noninferiority at 1.5 on the VAS. Alpha and beta risks were set at 2.5% and 20%, respectively. We assumed that the common SD was 1.9 based on our previous investigation in the clinic. Anticipating a dropout rate of 20%, we planned to recruit 34 patients in each group. The current sample size of 33 patients in each group achieved 88% statistical power.

## Results

Of the 545 patients with chronic cough who were being screened, 99 eligible patients meeting the inclusion criteria were randomly assigned to the M group (n = 33), BF group (n = 33) or MBF group (n = 33). As shown in Table 1, the subjects in these three groups were comparable in terms of demographics and baseline clinical characteristics (all p > 0.05). During the treatment period, 1 patient in the M group discontinued treatment due to an acute exacerbation at week 4, 2 patients in the BF group discontinued treatment due to ineffectiveness at week 1, and 3 patients in the MBF group discontinued treatment due to ineffectiveness or other reasons at week 4. (Fig. 2).

**Table 1:** Demographics and baseline characteristics

	M group (n = 33)	BF group (n = 33)	MBF group (n = 33)
Female, %	75.8	69.7	69.7
Age, yr	32.0 (27.5–52.0)	42.0 (33.5–51.0)	37.0 (29.0–54.5)
BMI	22.4 ± 3.2	22.7 ± 3.2	23.1 ± 3.2
Cough duration, mon	12.0 (3.8–36.3)	12.0 (4.0–40.5)	18.0 (3.5–67.0)
AR, %	50.0	57.6	51.5
Atopy, %	76.9	64.0	75.0
VAS	6.0 (5.0–7.0)	5.0 (5.0–9.0)	6.0 (5.0–8.0)
Daytime cough symptom score	2.0 (2.0–3.0)	2.0 (2.0–3.0)	2.0 (2.0–3.0)
Night-time cough symptom score	2.0 (1.0–3.0)	2.0 (1.0–3.0)	1.0 (1.0–2.0)
Log C5	1.2 (0.3–2.4)	1.8 (1.0–2.7)	1.8 (0.7–2.2)
FeNO	3.8 ± 0.6	4.1 ± 0.6	3.6 ± 0.6
Sputum Eos%	13.6 (2.0–37.6)	16.1 (4.5–31.5)	5.8 (1.3–14.5)
Blood Eos%	4.5 (2.1–7.2)	4.9 (3.3–8.4)	4.3 (3.0–7.4)
FEV_1_ pred%	93.3 ± 12.1	93.3 ± 12.1	94.6 ± 12.1
FEV_1_/FVC%	79.1 ± 8.0	78.5 ± 7.9	77.2 ± 8.0
MMEF%	64.1 ± 21.8	63.7 ± 21.5	56.3 ± 21.7
PD20	0.8 (0.3–2.4)	0.8 (0.2–2.2)	0.6 (0.2–1.8)

BMI, FEV_1_% pred, FEV_1_/FVC and MMEF% pred were expressed as mean ± SD; Age, cough duration, VAS, daytime cough symptom score, night-time cough symptom score, Log C5, sputum Eos%, blood Eos% and PD20 were expressed as median (IQR); FeNO was presented as geometric mean ± SD. Female, AR, Atopy were expressed as percentage. M: montelukast alone; BF: budesonide/formoterol alone; MBF: montelukast plus budesonide/formoterol. AR: allergic rhinitis. FVC: forced vital capacity; FEV_1_: forced expiratory volume in the first second; MMEF: maximal mid-expiratory flow

### Changes from baseline in the cough VAS score and cough symptom score

**Fig. 3:**
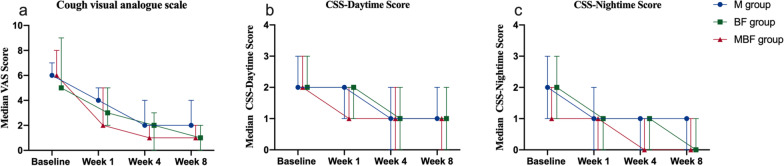
Median changes from baseline to week 1, week 4, and week 8 in VAS score (**a**), daytime CSS (**b**) and night-time CSS (**c**) of the patients in three groups. M: montelukast alone; BF: budesonide/formoterol alone; MBF: montelukast plus budesonide/formoterol; VAS: visual analog scale; CSS: cough symptoms score. Error bars indicate IQR

After 8 weeks of treatment, the M group showed a significant reduction in the median cough VAS (from 6.0 to 2.0, *p* < 0.001), daytime CSS (from 2.0 to 1.0, *p* < 0.001), and night-time CSS (from 2.0 to 1.0, *p* = 0.001). Likewise, the BF group and MBF group showed significant improvements in the VAS (from 5.0 to 1.0 in the BF group, *p* < 0.001; from 6.0 to 1.0 in the MBF group, *p* < 0.001), daytime CSS (from 2.0 to 1.0 in the BF group, *p* < 0.001; from 2.0 to 1.0 in the MBF group, *p* < 0.001), and night-time CSS (from 2.0 to 0.0 in the BF group, *p* < 0.001; from 1.0 to 0.0 in the MBF group, *p* < 0.001). No significant differences were found in these scores at any visit and the mean change from baseline to the 8-week visit among the three groups (all *p* > 0.05) (Table 2, Fig. 3).

**Table 2:** Main outcomes in the full analysis set after 8 weeks

	M group	BF group	MBF group
*Cough visual analogue scale (VAS)*	*n = 33*	*n = 33*	*n = 33*
Baseline, median (IQR)	6.0 (5.0, 7.0)	5.0 (5.0, 9.0)	6.0 (5.0, 8.0)
Week 1, median (IQR)	4.0 (2.0, 5.0) *	3.0 (2.0, 5.0) *	2.0 (1.0, 5.0) *
Week 4, median (IQR)	2.0 (1.0, 4.0) *	2.0 (0.0, 3.0) *	1.0 (0.0, 2.0) *
Week 8, median (IQR)	2.0 (1.0, 4.0) *	1.0 (0.0, 2.0) *	1.0 (0.0, 2.0) *
Week 1 mean change from baseline, mean (95%CI)	− 2.3 (− 3.1, − 1.5) p < 0.001	− 2.9 (− 3.7, − 2.1) p < 0.001	− 3.2 (− 4.0, − 2.4) p < 0.001
Week 4 mean change from baseline, mean (95%CI)	− 3.1 (− 3.9, − 2.3) p < 0.001	− 4.1 (− 4.9, − 3.3) p < 0.001	− 4.5 (− 5.3, − 3.7) p < 0.001
Week 8 mean change from baseline, mean (95%CI)	− 3.5 (− 4.3, − 2.7) p < 0.001	− 4.9 (− 5.7, − 4.1) p < 0.001	− 4.7 (− 5.5, − 3.9) p < 0.001
*Cough symptom score-daytime*	*n = 33*	*n = 33*	*n = 33*
Baseline, median (IQR)	2.0 (2.0, 3.0)	2.0 (2.0, 3.0)	2.0 (2.0, 3.0)
Week 1, median (IQR)	2.0 (1.0, 2.0) *	2.0 (1.0, 2.0) *	1.0 (1.0, 2.0) *
Week 4, median (IQR)	1.0 (0.0, 2.0) *	1.0 (0.0, 2.0) *	1.0 (0.0, 2.0) *
Week 8, median (IQR)	1.0 (1.0, 2.0) *	1.0 (0.0, 2.0) *	1.0 (0.0, 1.0) *
Week 1 mean change from baseline, mean (95%CI)	− 0.8 (− 1.1, − 0.4) p < 0.001	− 0.6 (− 1.0, − 0.2) P = 0.003	− 0.7 (− 1.1, − 0.3) p < 0.001
Week 4 mean change from baseline, mean (95%CI)	− 1.1 (− 1.5, − 0.7) p < 0.001	− 1.1 (− 1.5, − 0.7) p < 0.001	− 1.3 (− 1.7, − 0.9) p < 0.001
Week 8 mean change from baseline, mean (95%CI)	− 1.3 (− 1.7, − 0.9) p < 0.001	− 1.4 (− 1.8, − 1.0) p < 0.001	− 1.4 (− 1.8,1.0) p < 0.001
*Cough symptom score-night-time*	*n = 33*	*n = 33*	*n = 33*
Baseline, median (IQR)	2.0 (1.0, 3.0)	2.0 (1.0, 3.0)	1.0 (1.0, 2.0)
Week 1, median (IQR)	1.0 (0.0, 2.0) *	1.0 (0.0, 1.0) *	1.0 (0.0, 1.0) *
Week 4, median (IQR)	1.0 (0.0, 1.0) *	1.0 (0.0, 1.0) *	0.0 (0.0, 1.0) *
Week 8, median (IQR)	1.0 (0.0, 1.0) *	0.0 (0.0, 1.0) *	0.0 (0.0, 1.0) *
Week 1 mean change from baseline, mean (95%CI)	− 0.9 (− 1.3, − 0.5) p < 0.001	− 1.2 (− 1.54, − 0.78) p < 0.001	− 0.8 (− 1.2, − 0.4) p < 0.001
Week 4 mean change from baseline, mean (95%CI)	− 1.2 (− 1.6, − 0.8) p < 0.001	− 1.5 (− 1.9, − 1.1) p < 0.001	− 1.1 (− 1.5, − 0.7) p < 0.001
Week 8 mean change from baseline, mean (95%CI)	− 1.2 (− 1.6, − 0.8) p < 0.001	− 1.7 (− 2.1, − 1.3) p < 0.001	− 1.3 (− 1.7, − 0.9) p < 0.001

Values of VAS, CSS were presented as median (IQR); Values of the change in VAS, CSS were presented as mean (95%CI). M: montelukast alone; BF: budesonide/formoterol alone; MBF: montelukast plus budesonide/formoterol. VAS: visual analog scale; CSS: cough symptom score. VS baseline: ^#^, *p* < 0.05; *, *p* < 0.01

### Change from baseline in the cough reflex sensitivity

**Fig. 4:**
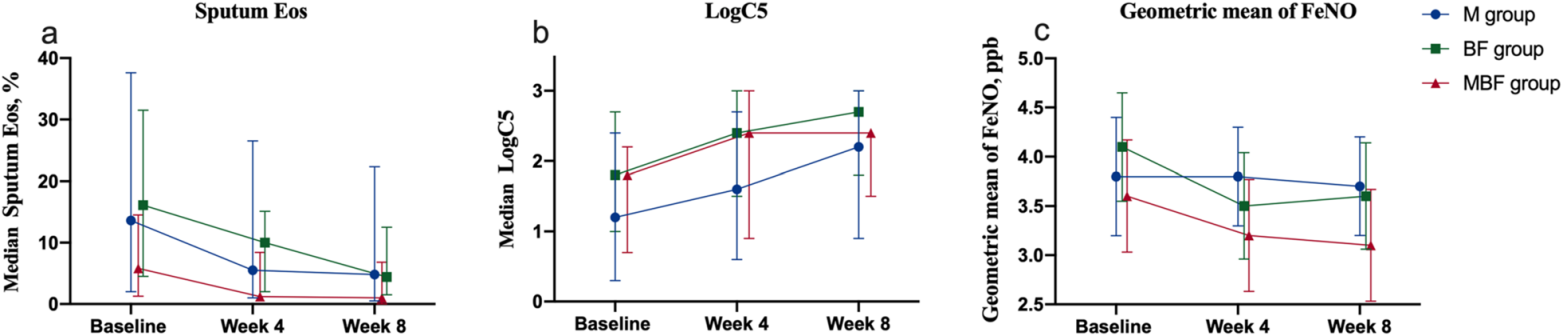
Median changes from baseline to week 4, and week 8 in LogC5 (**a**) and sputum Eos% (**b**), and mean changes from baseline in FeNO (**c**) of the patients in three groups. FeNO values were logged. M: montelukast alone; BF: budesonide/formoterol alone; MBF: montelukast plus budesonide/formoterol. Error bars indicate IQR or SD

The results for the secondary endpoints revealed the treatment efficacies of all three groups. For cough reflex sensitivity, the M group showed improvement in the LogC5 (median level from 1.2 at baseline to 2.2 at week 8, *p* = 0.017), with a mean change of 0.6 (95% CI: 0.1–1.0). Improvement in the LogC5 was found in the BF group (from 1.8 to 2.7, *p* = 0.033) and the MBF group (from 1.8 to 2.4, *p* = 0.019). The mean change from baseline was 0.5 (95% CI: 0.0–0.9) in the BF group and 0.5 (95% CI: 0.1–1.0) in the MBF group. The cough reflex sensitivity among the three groups at all visits and the mean change from baseline to the week 8 visit were comparable (all *p* > 0.05) (Table 3, Fig. 4).

**Table 3:** Secondary Outcomes in the Full Analysis Set After 8 Weeks

LogC5	M group	BF group	MBF group
Baseline, median (IQR)	1.2 (0.3, 2.4) (n = 31)	1.8 (1.0, 2.7) (n = 32)	1.8 (0.7, 2.2) (n = 32)
Week 4, median (IQR)	1.6 (0.6, 2.7) (n = 28)	2.4 (1.5, 3.0) (n = 29)	2.4 (0.9, 3.0) (n = 32)
Week 8, median (IQR)	2.2 (0.9, 3.0) ^#^ (n = 30)	2.7 (1.8, 3.0) ^#^ (n = 31)	2.4 (1.5, 2.4) ^#^ (n = 32)
Week 4 mean change from baseline, mean (95%CI)	0.3 (− 0.2, 0.8) (n = 28) p = 0.234	0.3 (− 0.1, 0.8) (n = 29) p = 0.170	0.5 (0.1, 1.0) (n = 32) p = 0.064
Week 8 mean change from baseline, mean (95%CI)	0.6 (0.1, 1.0) (n = 30) p = 0.017	0.5 (0.0, 0.9) (n = 31) p = 0.033	0.5 (0.1, 1.0) (n = 32) p = 0.019
*Sputum eosinophil, %*			
Baseline, median (IQR)	13.6 (2.0, 37.6) (n = 32)	16.1 (4.5, 31.5) (n = 32)	5.8 (1.3, 14.5) (n = 32)
Week 4, median (IQR)	5.5 (1.0, 26.5) * (n = 29)	10.0 (2.0, 15.1) (n = 30)	1.2 (0.5, 8.4) (n = 28)
Week 8, median (IQR)	4.8 (0.5, 22.3) * (n = 31)	4.4 (1.5, 12.5) ^#^ (n = 30)	1.0 (0.0, 6.8) ^#^ (n = 31)
Week 4 median change from baseline, median (IQR)	− 4.3 (− 18.5, − 0.3) (n = 29) p = 0.002	− 3.6 (− 22, 0.5) (n = 30) p = 0.413	− 4.3 (− 23.0, 2.5) (n = 28) p = 0.373
Week 8 median change from baseline, median (IQR)	− 1.5 (− 29.8, 0.3) (n = 31) p = 0.012	− 4.3 (− 23.0, 2.5) (n = 30) p = 0.050	− 3.0 (− 9.0, − 0.5) (n = 31) p = 0.023
*Geometric mean of FeNO, ppb*			
Baseline, mean (SD)	3.8 ± 1.0 (n = 31)	4.1 ± 0.8 (n = 30)	3.6 ± 0.9 (n = 32)
Week 4, mean (SD)	3.7 ± 0.8 (n = 30)	3.6 ± 0.5 * (n = 29)	3.1 ± 0.7 * (n = 32)
Week 8, mean (SD)	3.7 ± 0.8 (n = 30)	3.5 ± 0.7 * (n = 29)	3.1 ± 0.7 * (n = 32)
Week 4 mean change from baseline, mean (95%CI)	− 0.1 (− 0.5, 0.2) (n = 30) p = 0.416	− 0.6 (− 0.9, − 0.2) (n = 29) p = 0.002	− 0.5 (− 0.8, − 0.1) (n = 32) p = 0.009
Week 8 mean change from baseline, mean (95%CI)	− 0.1(− 0.5, 0.2), (n = 30) p = 0.457	− 0.6 (− 1.0, − 0.3) (n = 29) p = 0.001	− 0.5 (− 0.8, − 0.1) (n = 32) p = 0.008

Values of LogC5, sputum Eos% and change of sputum Eos% were presented as median (IQR); FeNO was presented as geometric mean. Values of the change in LogC5, FeNO were presented as mean (95%CI) and the change of sputum Eos% was presented as median (IQR). M: montelukast alone; BF: budesonide/formoterol alone; MBF: montelukast plus budesonide/formoterol. FeNO: fractional exhaled nitric oxide; VS baseline: ^#^, *p* < 0.05; *, *p* < 0.01

### Change from baseline in the sputum Eos% and FeNO

In terms of airway inflammation, all three groups showed significant decreases in sputum Eos% as compared with the baseline (median from 13.6% to 4.8% in the M group, *p* = 0.012; 16.1% to 4.4% in the BF group, *p* = 0.050; from 5.8% to 1.0% in the MBF group, *p* = 0.023). No significant differences in the change of sputum Eos% from baseline to week 8 were observed among the three groups (all *p* > 0.05) (Table 3, Fig. 4). Additionally, the rate of sputum Eos% decreasing to a normal level (< 2.5%) in the MBF group was significantly higher than that in the M group (52.2% vs 16.0%, p < 0.05) and BF group (52.2% vs 30.8%, p < 0.05), respectively. In addition, the M group did not show a significant decrease in FeNO levels at week 8 as compared with the baseline (geometric mean from 3.8 to 3.7, *p* = 0.140). The FeNO levels of the BF group and MBF group decreased significantly from baseline to week 8 of treatment (BF group, 4.1 to 3.6, *p* = 0.001; MBF group, from 3.6 to 3.1, *p* = 0.008). A significantly lower FeNO level at week 8 was found in the MBF group than in the M group and the BF group (all *p* < 0.05), respectively, and a larger improvement in the FeNO level from baseline to week 8 was shown in the MBF group and BF group than in the M group (all *p* < 0.05) (Table 3, Fig. 4).

### Other outcomes

After 8 weeks of treatment, significant improvements in the FEV_1_/FVC ratio (mean level from 77.2% to 79.5%), MMEF% (56.3% to 65.2%) and percentages of blood eosinophils (median level from 4.3% to 3.5%) were found only in the MBF group as compared to the baseline, respectively (all *p* < 0.01) (Additional file 1: Table S1).

According to the patients’ reports, a remarkably shorter trend of the median onset time of cough improvement was found in the MBF group [24.0 h (IQR: 13.0–72.0)] as compared with the BF group [48.0 h (24–144)] and the M group [74.0 h (IQR: 24.0–168.0)], although no significant differences were observed. No serious adverse events were reported in the patients receiving any treatment.

### Follow-up after the treatment: cough relapse and wheezing development

Finally, a total of 87 patients completed the 8-week follow-up after the 8-week treatment period, with 29 patients in every treatment group respectively, among whom, wheezing was reported by 2 (6.9%) patients in the M group and 1 (3.4%) patient in the BF group, while no patients in the MBF group reported the occurrence of wheezing. After the 8-week treatment period, 74 patients (22 in the M group, 26 in the BF group and 26 in the MBF group) who showed a complete cough relief or only experienced an occasional cough discontinued asthmatic treatment during follow-up. Among these 74 patients, cough relapse occurred in 6 (27.3%) patients in the M group, 8 (30.8%) patients in the BF group and 5 (19.2%) patients in the MBF group during the 8-week follow-up. However, no significant differences were found in the rate of cough relapse and progression to wheezing among the three groups. A long-term follow-up is needed to determine whether different treatments play a role in the prognosis of the patients with CVA.

## Discussion

In this study, we first investigated and compared the efficacy of LTRAs, ICS/LABA and LTRAs plus ICS/LABA in the treatment of CVA. We found that montelukast alone could effectively improve the cough VAS score, daytime and night-time cough symptoms scores, and capsaicin cough sensitivity, and decrease sputum eosinophil counts. Furthermore, the montelukast alone showed antitussive and anti-inflammatory efficacies similar to those of ICS/LABA or montelukast plus ICS/LABA. In addition, patients who received the combination treatment of montelukast plus ICS/LABA demonstrated a significant improvement in lung function parameters and peripheral eosinophils.

According to our results, no significant differences in the improvement of the cough VAS score, daytime CSS or night-time CSS among the three groups were found. More importantly, our results suggested that the antitussive effect of montelukast alone on CVA was similar to that of budesonide/formoterol alone and the combination of both, indicating that LTRAs may offer an alternative for children or elderly patients who are unable to master inhalation methods or subjects who are unable to use ICS because of the side effects. However, the onset time of cough improvement in the MBF group was relatively shorter than that in the M group, and a visually shorter trend was shown in the BF group than in the M group. The quick effect of LABA might underlie the differences. Our data showed that cough improvement occurred in parallel to the decrease of the sputum eosinophils and that montelukast administration alone could effectively relieve airway eosinophilic inflammation at both week 4 and week 8, which was consistent with previous reports [14, 23]. However, this study (Additional file 1: Table S2) and previous studies did not find significant correlations between the improvement of cough symptoms (VAS score, LogC5) and the change in sputum eosinophils [14, 24]. Recent studies have shown that eosinophils can interact with airway sensory nerve fibres in asthmatics and promote increased airway sensory fibre density and nerve remodeling, which may be involved in the pathogenesis of airway hyper-responsiveness and cough hypersensitivity [25–28]. The decreased eosinophilic inflammation did not correlate with the improvement of cough, suggesting that besides eosinophils, other immune cells may also play important roles in the pathophysiology of cough in CVA patients. Seriko Kawai et al. studied the clinical effects of montelukast in CVA patients and compared the difference of mast cells in bronchial mucosa biopsy specimens of the montelukast-responsive/unresponsive groups. Responsive CVA patients showed a higher proportion of CD63-positive cells in tryptase-positive mast cells in the bronchial mucosa biopsy specimens than unresponsive patients, suggesting that the activation of airway mast cells may be an essential feature in montelukast-responsive CVA patients [29]. Given the evidence that CysLTs were mainly synthesized from mast cells and eosinophils [30]. It suggested that mast cell as well as eosinophils-induced CysLTs can contribute to the mechanism of CVA.

Consistent with previous studies, our results showed that 8 weeks of treatment with montelukast alone could also significantly decrease the cough reflex sensitivity in CVA patients, but the spirometry function remained unchanged, which further suggested that montelukast could relieve cough by decreasing cough reflex sensitivity rather than by improving bronchoconstriction. Dicpinigaitis et al. reported that zafirlukast might exert a therapeutic role in CVA by inhibiting cough sensitivity [12]. Interestingly, the expression of substance P (SP) in the airway was increased in CVA patients compared with classic asthmatic patients and healthy subjects [31], while CysLTs stimulated the release of SP from neurons [32, 33]. Takemura M et al. demonstrated that 4 weeks of treatment with the LTRA montelukast did not affect the sputum levels of mediators, including CysLTs [14]. Thus, LTRAs decreased cough reflex sensitivity by reducing the release of SP, which might be biologically plausible.

Our study showed that the improvement of cough precedes objective changes in sputum eosinophils or cough sensitivity. Although our current data did not reflect the objective changes in sputum eosinophils or cough sensitivity in the first week, the changes of sputum eosinophil percentages and LogC5 at week 4 indicated that the period to reach a significant change in sputum eosinophils or cough sensitivity should be longer. This was in consistent with the common clinical phenomenon that symptoms improve but still present abnormal parameters. Takemura et al. demonstrated that 4 weeks of treatment with the LTRA montelukast did not affect the sputum levels of mediators, including CysLTS [14]. Therefore, it was speculated that the persistently higher sputum levels of CysLTS stimulated the release of substance P, which was attributed to the lagging effect of objective changes compared with subjective improvement of cough. Moreover, at week 8, both cough sensitivity and sputum eosinophils improved significantly, suggesting that the 8 weeks may be the optimum course of treatment in the management of patients with CVA, which needs to be confirmed in further study.

LTRAs added to ICS have been reported to provide greater efficacy than ICS alone in the treatment of classic asthma [34]. For CVA patients, LTRAs were recommended as an add-on treatment or stepping-up treatment if cough symptom remained following ICS treatment [10, 11]. In our study, we did not find clear superiority in the improvement of cough symptom, cough reflex sensitivity or sputum Eos% with the treatment of montelukast plus budesonide/formoterol, indicating that the combination treatment seems to be unnecessary for CVA patients. The mechanism underlying the different efficacy of LTRAs in the treatment of CVA and classic asthma is unclear. Tajiri et al. found that the sputum eosinophil proportion and FeNO in classic asthma patients were higher than those in CVA patients [35] and that the different levels of airway eosinophilic inflammation in CVA and classic asthma patients might result in the different efficacy of LTRAs. Thus, we speculated that CysLTs might play a more significant role in the pathogenesis of cough and eosinophilic airway inflammation in CVA.

In addition, we noticed that cough was not completely relieved in 10 patients after 8 weeks of treatment. Among these 10 patients, cough was relieved completely in 3 patients after another 4 weeks of anti-asthmatic treatment, which suggested that cough in a small number of CVA patients might not be controlled well by for a treatment period of only 8 weeks. The cough guidelines released by the ACCP, the ERS, and China suggested that CVA should be treated for at least 8 weeks [15, 16, 36]. Meanwhile, we found that sputum eosinophilia persisted in more than 60% of the patients receiving ICS/LABA and approximately 50% of the patients receiving the combination treatment, although cough symptoms were relieved in most of the patients with these treatments, which indicated that 8 weeks of treatment with ICS/LABA or combination with LTRA was far more from enough to improve airway inflammation in CVA patients. Currently, the optimal length of the antiasthma treatment for CVA patients is uncertain, and further investigation is needed.

During the follow-up period after the study, we found a lower trend of cough relapse rate in the combination treatment group than in the other two monotherapy groups with monotherapy. Moreover, no patient in this group reported a progression to wheezing. Sputum eosinophilia has been reported to be a risk factor for progression to classic asthma in CVA [37]. According to our results, patients treated with the combination treatment of montelukast plus ICS/LABA showed a significantly lower ratio of patients with sputum eosinophilia and a significant decrease in blood eosinophils after 8 weeks of treatment, while no differences were observed in the cough score, LogC5 or lung function among the three groups. Thus, we speculated that the improvement of airway inflammation, rather than a complete relief of cough, might be more likely be related to the prognosis of CVA in the short term, but a well-designed study is needed to confirm this hypothesis in the future. Furthermore, whether a combination treatment of LTRAs plus ICS/LABA may lead to a better prognosis of CVA than LTRAs alone or ICS/LABA alone in the long term also needs to be established.

Some limitations in our study should be noted. First, we did not apply the objective measurements, such as cough frequency records, to assess the efficacy of treatment because it was still not available for clinic trial in China when this study began. Thus, we had to choose the subjective tools, including the VAS and CSS instead. We strictly followed the minimal significant improvement of cough as a change in the VAS of 15 cm according to the British Thoracic Society guidelines of cough [36]. The overall effective rate in our study (data not shown) was consistent with that reported in a study by Spector et al. [13], which assessed cough via audio cough recordings. Second, although there was a lack of a placebo group in our study that might have affected the precise evaluation of treatment effects, previous relevant placebo-control studies showed the effectiveness of montelukast in patients with CVA [12, 13]. In addition, a placebo-controlled study would be worth conducting in the future. Third, we added a preliminary data on the prognosis of patients with CVA. However, our study had a short-term follow-up period of only 8 weeks, and a longer follow-up duration is needed.

## Conclusions

In conclusion, montelukast can effectively improve cough symptoms, cough reflex sensitivity and eosinophilic airway inflammation in patients with CVA. The antitussive and anti- inflammatory efficacies are similar to those of budesonide/formoterol alone and the combination of both. Thus, monotherapy with montelukast may be a good therapeutic alternative for patients with CVA. A combination treatment of budesonide/formoterol plus montelukast leads to additional improvements in lung function and the alleviation of peripheral eosinophils. Whether the combination treatment plays a role in the long-term prognosis of CVA needs to be further investigated.

## Supplementary Information

**Additional file 1. S-Table1**. Other outcomes in the Full Analysis Set after 8 Weeks.** S-Table2**. Correlations between sputum Eos% and cough VAS, Log C5 in montelukast group

## Data Availability

The data and/or related materials of this study are available from the corresponding author on reasonable request.

## Abbreviations

CVA: Cough variant asthma
CSS: Cough symptom score
CRS: Cough reflex sensitivity
CysLTs: Cysteinyl leukotrienes
FeNO: Fractional exhaled nitric oxide
ICS: Inhaled corticosteroids
LABA: Long-acting β2-agonist
LTRAs: Cysteinyl-leukotrienes receptor antagonists
SP: Substance P
VAS: Cough visual analog scale
