# Supplementary material for: Effects of treatment with montelukast alone, budesonide/formoterol alone and a combination of both in cough variant asthma
Source: Respir Res. 2022 Oct 10;23:279. doi: 10.1186/s12931-022-02114-6 (PMC9552469; doi:10.1186/s12931-022-02114-6)
Supplement: Supplementary file 1 — Additional file 1. S-Table1. Other outcomes in the Full Analysis Set after 8 Weeks. S-Table2. Correlations between sputum Eos% and cough VAS, Log C5 in montelukast group [file 12931_2022_2114_MOESM1_ESM.docx]

**Supplementary file**

**Supplementary table**

**S-Table1 Other outcomes in the Full Analysis Set after 8 Weeks**

| **Blood Eosinophil, %** | M group | BF group | MBF group |
| --- | --- | --- | --- |
| Baseline, median (IQR) | 4.5 (2.1, 7.2)  (n=30) | 4.9(3.3, 8.4)  (n=28) | 4.3(3.0, 7.4)  (n=31) |
| Week 8, median (IQR) | 4.7 (2.9, 6.7)  (n=31) | 4.4(2.2, 5.7)  (n=25) | 3.5(2.2, 4.3)  (n=29) |
| Week 8 median change from baseline, median (IQR) | -0.6 (-4.6, 5.5)  (n=30) p=0.150 | -0.14 (-6.7, 5.3)  (n=25) p=0.121 | -1.6(-2.9, -0.2) *  (n=29) p=0.013 |
| **FEV_1_/FVC, %** |  |  |  |
| Baseline, mean (SD) | 79.1±8.0  (n=33) | 78.5±7.9  (n=33) | 77.2±8.0  (n=33) |
| Week 8, mean (SD) | 80.0±8.0  (n=33) | 80.0±7.9  (n=32) | 79.5±7.7 ^#^  (n=30) |
| Week 8 mean change from baseline, mean (95%CI) | 0.9 (-1.2, 3.1)  (n=33) p=0.388 | 1.5 (-0.7, 3.7)  (n=32) p=0.172 | 2.4 (0.16, 4.6)  (n=30) p=0.036 |
| **MMEF pred, %** |  |  |  |
| Baseline, mean (SD) | 64.1±21.8  (n=33) | 63.7±21.5  (n=33) | 56.3±21.7  (n=33) |
| Week 8, mean (SD) | 63.6±21.8  (n=33) | 68.4±21.5  (n=32) | 65.2±21.4 *  (n=30) |
| Week 8 mean change from baseline, mean (95%CI) | -0.57 (-5.9, 4.7)  (n=33) p=0.832 | 4.7 (-0.8, 10.1)  (n=32) p=0.094 | 8.9 (3.2, 14.6)  (n=30) p=0.002 |
| **FEV_1_pred, %** |  |  |  |
| Baseline, mean (SD) | 93.3±12.1  (n=33) | 93.3±12.1  (n=33) | 94.6±12.1  (n=33) |
| Week 8, mean (SD) | 93.4±12.1  (n=33) | 96.0±12.1  (n=30) | 96.4±12.1  (n=31) |
| Week 8 mean change from baseline, mean (95%CI) | 0.05 (-2.8, 2.9)  (n=33) p=0.969 | 2.69 (-0.3, 5.6)  (n=30) p=0.073 | 1.78 (-1.1, 4.7)  (n=31) p=0.229 |

Blood Eos% was presented as median (IQR); FEV_1_/FVC, FEV_1_pred%, MMEF pred% were presented as mean±SD; Values of the change in blood Eos% was presented as median (IQR); Values of the change in FEV_1_/FVC, FEV_1_pred%, MMEF pred% were presented as mean (95%CI). Abbreviations: M: montelukast alone; BF: budesonide/formoterol alone; MBF: montelukast plus budesonide/formoterol. FVC: forced vital capacity; FEV_1_: forced expiratory volume in the first second MMEF: maximal mid-expiratory flow. VS baseline: ^#^, *p*<0.05; *, *p*<0.01.

**S-Table2 Correlations between sputum Eos% and cough VAS, Log C5 in montelukast group**

|  | | r | p |
| --- | --- | --- | --- |
| Sputum Eos%  (Baseline) | VAS  (Baseline) | -0.199 | 0.274 |
|  | LgC5  (Baseline) | 0.132 | 0.487 |
| Sputum Eos%  (Week 8) | VAS  (Week 8) | -0.088 | 0.637 |
|  | LgC5  (Week 8) | 0.039 | 0.838 |
| △Sputum Eos% | △VAS | 0.027 | 0.884 |
|  | △LgC5 | 0.117 | 0.546 |
